# Optimization of Spatial and Temporal Configuration of a Pressure Sensing Array to Predict Posture and Mobility in Lying

**DOI:** 10.3390/s23156872

**Published:** 2023-08-02

**Authors:** Silvia Caggiari, Liudi Jiang, Davide Filingeri, Peter Worsley

**Affiliations:** 1 Skin Sensing Research Group, School of Health Sciences, Faculty of Environmental and Life Sciences, University of Southampton, Southampton SO17 1BJ, UK; d.filingeri@soton.ac.uk (D.F.); p.r.worsley@soton.ac.uk (P.W.); 2 School of Engineering, Faculty of Engineering and Physical Sciences, University of Southampton, Southampton SO17 1BJ, UK; l.jiang@soton.ac.uk

**Keywords:** high-resolution pressure sensing arrays, pressure ulcers, posture and mobility, optimized configuration, receiver operating characteristic curve, convolutional neural network

## Abstract

Commercial pressure monitoring systems have been developed to assess conditions at the interface between mattress/cushions of individuals at risk of developing pressure ulcers. Recently, they have been used as a surrogate for prolonged posture and mobility monitoring. However, these systems typically consist of high-resolution sensing arrays, sampling data at more than 1 Hz. This inevitably results in large volumes of data, much of which may be redundant. Our study aimed at evaluating the optimal number of sensors and acquisition frequency that accurately predict posture and mobility during lying. A continuous pressure monitor (ForeSitePT, Xsensor, Calgary, Canada), with 5664 sensors sampling at 1 Hz, was used to assess the interface pressures of healthy volunteers who performed lying postures on two different mattresses (foam and air designs). These data were down sampled in the spatial and temporal domains. For each configuration, pressure parameters were estimated and the area under the Receiver Operating Characteristic curve (AUC) was used to determine their ability in discriminating postural change events. Convolutional Neural Network (CNN) was employed to predict static postures. There was a non-linear decline in AUC values for both spatial and temporal down sampling. Results showed a reduction of the AUC for acquisition frequencies lower than 0.3 Hz. For some parameters, e.g., pressure gradient, the lower the sensors number the higher the AUC. Posture prediction showed a similar accuracy of 63−71% and 84−87% when compared to the commercial configuration, on the foam and air mattress, respectively. This study revealed that accurate detection of posture and mobility events can be achieved with a relatively low number of sensors and sampling frequency.

## 1. Introduction

There are many situations in both hospital and community settings where individuals spend prolonged periods in beds or chairs as a result of restricted mobility and impaired sensation. This can result in the breakdown of skin and soft tissues, typically over bony prominences, commonly termed pressure ulcers (PUs) [1]. PUs represent a major burden to populations worldwide, with a significant impact on the quality of life of the individuals affected [2]. In the United Kingdom, their treatment is estimated to cost the National Health System (NHS) approximately £8 billion p.a. [3].

For several decades, pressure sensing arrays have been employed as visual feedback to assess the conditions at the support surface interface in both lying and sitting to optimize repositioning and prescribe effective mattresses and cushions, thus protecting the most vulnerable individuals [4]. This has been typically performed either at a single time point or averaged over relatively short time periods, providing a “snapshot” of the interface conditions. However, the interpretation of these data can at best provide a limited overview of the long-term effects of posture and mobility.

International and national guidelines recommend a generic repositioning frequency of approximately 2−4 h in bed and more frequently in a chair [1]. However, there is strong evidence that this frequency of movement is not adhered to in specific sub-patient groups, e.g., spinal cord injury patients [5]. Therefore, effective monitoring posture and mobility is critical for PU prevention and could support clinical decision making and personalized intervention in an objective manner.

Where appropriately adapted, pressure sensing technologies have the potential to monitor over extended periods to enable temporal evaluation of the interface conditions [6], and we have recently demonstrated their long-term use as a surrogate for posture and mobility detection [7]. Specific parameters estimated from the pressure data, such as center of pressure (COP) and contact area, were sensitive and specific to postural change events, and Convolutional Neural Network (CNN) was employed to predict static postures [8]. This was successfully translated for vulnerable patients, e.g., Spinal Cord Injured during prolonged periods of lying and sitting [5,9].

Commercial pressure monitoring systems are typically manufactured with more than 1000 sensors sampling data at high frequencies e.g., >1 Hz. This results in a large volume of data, which is complex to interpret, inevitably resulting in substantive redundancy [10,11]. The acquirement of large and non-relevant data sets place constraints on data transfer and data processing, hindering efficient and real-time feedback and diagnosis, limiting their accessibility and affecting adaptability. In addition, they have high costs (up to £20,000), which also limits their accessibility to a range of clinical settings, e.g., private homes, residential care, and nursing homes. Thus, there is a critical need to improve their design in terms of spatial and temporal resolution to reduce the computational cost derived from the acquisition and storage of large and redundant data and the economic cost to make these technologies accessible to a variety of settings. Software and IoT technologies are required to analyze pressure data acquired for long time periods as well as feedback patients, careers, and healthcare providers.

Literature reports several studies [12,13] assessing the optimal sensors’ distribution to accurately predict seated postures. However, these analytical methods have been limited to assess the optimal spatial resolution and predict postures from pressure data acquired on one type of cushion, e.g., foam. A recent study [14] extended this methodology to lying environment to assess an optimal temporal resolution that would detect postural change events. However, this was limited to detecting time-dependent changes in the distribution of pressure values and did not extend to predicting posture.

The present study aims to optimize sensor configuration for lying posture prediction in both the spatial and temporal domain. This will be achieved through an off-line sensitivity analysis of pressure values, down sampling from a high resolution and high frequency acquisition of different lying postures on two different mattresses (i.e., foam and air designs) which represent the widely used type of support surface in hospital and community settings. The down-sampled data will be used to predict movement events and postures based on validated algorithms [5,9]. An optimal spatial and temporal configuration that predicts posture and mobility events with a similar accuracy to a commercial high-resolution array will be evaluated and discussed.

## 2. Materials and Methods

### 2.1. Experimental Pressure Data

We used a set of experimental data derived from different studies on able-bodied volunteers who adopted a series of lying postures on different mattress systems, namely a castellated foam mattress (Medstrom, Ashby de la Zouche, Castle Donington, UK) and dynamic air cell mattresses (CellUNO, Care of Sweden, Tranemo, Sweden; and Virtuoso, Linet, Slaný, Czech Republic). Given that the dynamic mattresses were characterized by similar air cell designs, the corresponding experimental data were collated to form a single data set.

The studies on both foam and air cell mattress were conducted under institutional ethics (ERGO 26379 and 19647, respectively), and they involved 20 and 27 individuals (11M and 9F and 15M and 12F), respectively. Participants’ anthropometrics are reported in Table 1.

**Figure 1:**
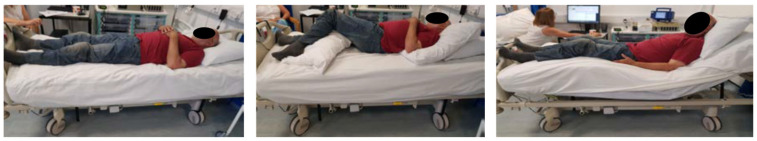
Images of the sagittal and lateral postures on the air cell mattress: Supine (**left**), right lateral turning (**middle**), 40° increment of the HOB (**right**).

Each participant was asked to adopt a series of sagittal and lateral lying postures on their corresponding mattress (Figure 1). Sagittal postures involved supine, high sitting (HS) with the head of the bed (HOB) set at 40°, and supine again. On the foam mattress, lateral posture was achieved with a continuous lateral rotational system (CLRS) (Vikta Komfitilt^®^, Pressure Care Management LTD, Andover, UK), placed underneath the mattress to evoke 20–25° lateral tilting, whilst on the air cell mattress, lateral postures were performed by placing pillows under the back and legs, in a similar manner to that adopted in clinical settings to off-load the sacrum through a 30° tilt [15].

Interface pressures were continuously monitored during each posture, using a high-resolution sensing array (ForeSite PT, XSensor, Calgary, Canada), with an acquisition frequency of 1 Hz [7] The mat incorporates 5664 pressure measuring sensor elements (118 × 48), with a spatial resolution of 15.9 mm, covering a sensing area of 762 mm × 1880 mm. Each sensor operates within a range of 5–200 mmHg (0.7–26.6 kPa) and an accuracy of ± 2 mmHg.

### 2.2. Data Analysis

**Figure 2:**
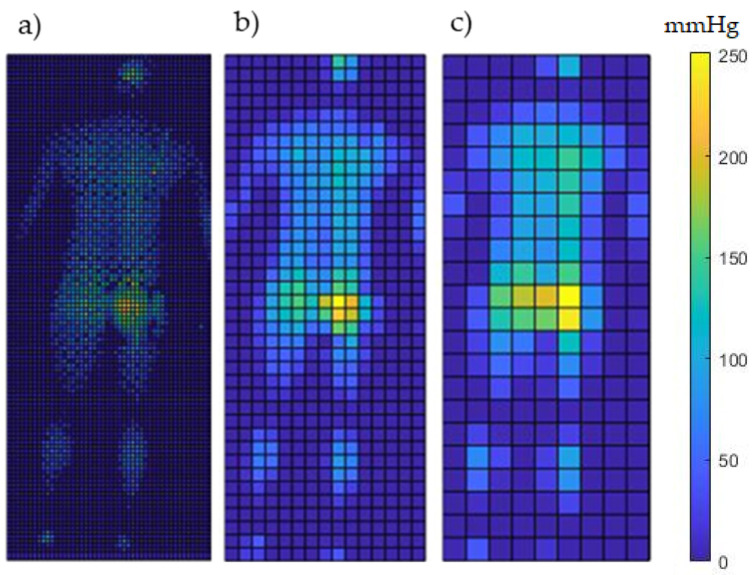
A depiction of a lying posture at different spatial resolutions represented through (**a**) the original resolution (5564 sensors); (**b**) data points aggregated into 3 × 3 regions, which resulted in 624 sensors; (**c**) data points aggregated into 5 × 5 regions, which resulted in 207 sensors.

Analysis for both data sets involved the same steps, which are explained below:

**Down sample the spatial and temporal resolution:** A series of coarser spatial configurations were obtained by combining regions of 2 × 2, 3 × 3, 4 × 4, and 5 × 5 sensors and averaging their values. This resulted in four different *spatial resolutions*, namely 1416 (59 × 24), 624 (39 × 16), 348 (29 × 12) and 207 (23 × 9) sensors, respectively (Figure 2).

For each of these resolutions, pressure data were down sampled in their frequency, considering 1 sample every 2, 3, 5, and 10 s, which resulted in *temporal resolutions* of 0.5, 0.3, 0.2, and 0.1 Hz, respectively. Table 2 summarizes the spatial and temporal configurations.

**Estimate a series of temporal pressure parameters:** For each of the spatial and temporal configurations, a series of parameters was estimated from the pressure distribution [7]. To briefly review, these involved:

- Center of pressure (COP), defined as the centroid of the distribution, in the longitudinal and transverse direction with respect to the long axis of the mat;
- Contact area between the mattress and the individuals, in which sensors recorded a pressure of or above a minimum threshold of 5, 10, and 20 mmHg;
- Peak pressure, which described the maximum pressure value;
- Peak pressure gradient, which described the maximum change in pressure between adjacent sensing cells.

**Figure 3:**
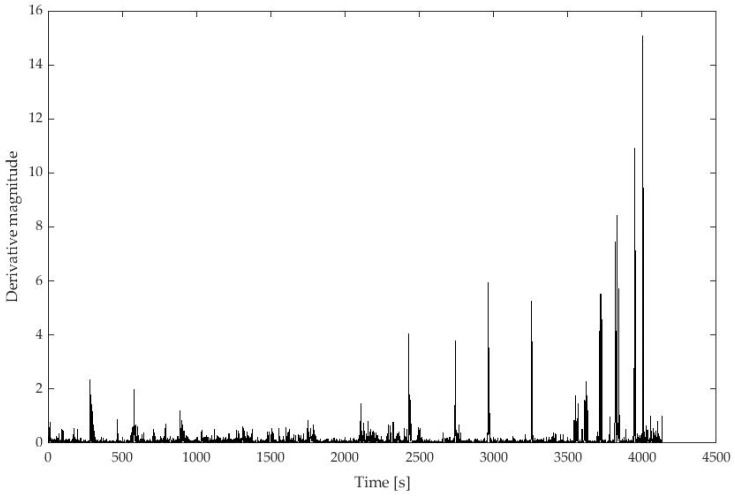
Derivative profile of center of pressure estimated in the longitudinal direction on a foam mattress with a spatial resolution of 5664 sensors. Each data point in the magnitude corresponds to the difference of COP magnitude at consecutive time points. Peak magnitudes are representative of postural changes events.

**Signal processing to assess the accuracy in discriminating between postural change events:** For all combinations of spatial and temporal configurations, the 1st spatial derivative of the pressure parameters was examined. The derivative represents the difference in the signal magnitude between consecutive time points and allows transitions between static postures to be identified, namely postural change events, through peak magnitudes [8]. Figure 3 shows the derivative profile of the center of pressure estimated in the longitudinal direction on the air cell mattress, for a spatial configuration of 5664 sensors.

A Receiver Operating Characteristic (ROC) analysis was then performed within SPSS v27 (IBM SPSS Statistics, Armonk, NY, USA) to determine the accuracy of the parameters for each spatial and temporal configurations to discriminate between the presence and absence of postural changes. The area under the ROC curve (AUC), which plots sensitivity (true positive) versus false positive rate (100–specificity) [7], was calculated to assess the overall accuracy of each parameter.

**Convolutional Neural Network (CNN) to predict static postures:** For all the spatial resolutions, the pressure distribution associated with the static postures was converted into grey scale images. These were then utilized for prediction of specific postures (supine, lateral lying, and high sitting) using the CNN [9]. For each spatial configuration, a training model was generated with 80% of the image data for each of the participants, and the accuracy in classifying the postures was then assessed with the remaining 20%, which represented the test data set.

## 3. Results

### 3.1. Postural Movement Events: ROC Analysis

Table 3 summarizes the AUC values to predict postural movement events associated with all parameters and their corresponding spatial and temporal configuration, for both the foam and air cell mattress. Our analysis showed that the AUC associated with contact area calculated at thresholds of 5, 10, and 20 mmHg was approximately similar, for all the spatial and temporal configurations. Therefore, the current analysis focus on AUC values associated with contact area was estimated at a threshold ≥ 20 mmHg [7].

The findings from ROC analysis revealed a higher accuracy in detecting postural events for the air cell mattress compared to the foam mattress. On both mattress conditions, the parameters showed that AUC values were approximately similar at a sampling frequency ranging between 1 and 0.3 Hz for all spatial resolutions, with a steady decline for frequencies lower than 0.3 Hz. In some parameters, for example peak pressure, this decrease ranged between 10 and 20%. At each sampling frequency, down sampling the number of sensors did not lead to relevant changes in the AUC values for the COP estimated on the foam mattress. By contrast, its counterpart on the air cell mattress showed the highest AUC at a spatial resolution of 5664 sensors, with this value decreasing ≤ 10% for lower spatial resolutions.

**Figure 4:**
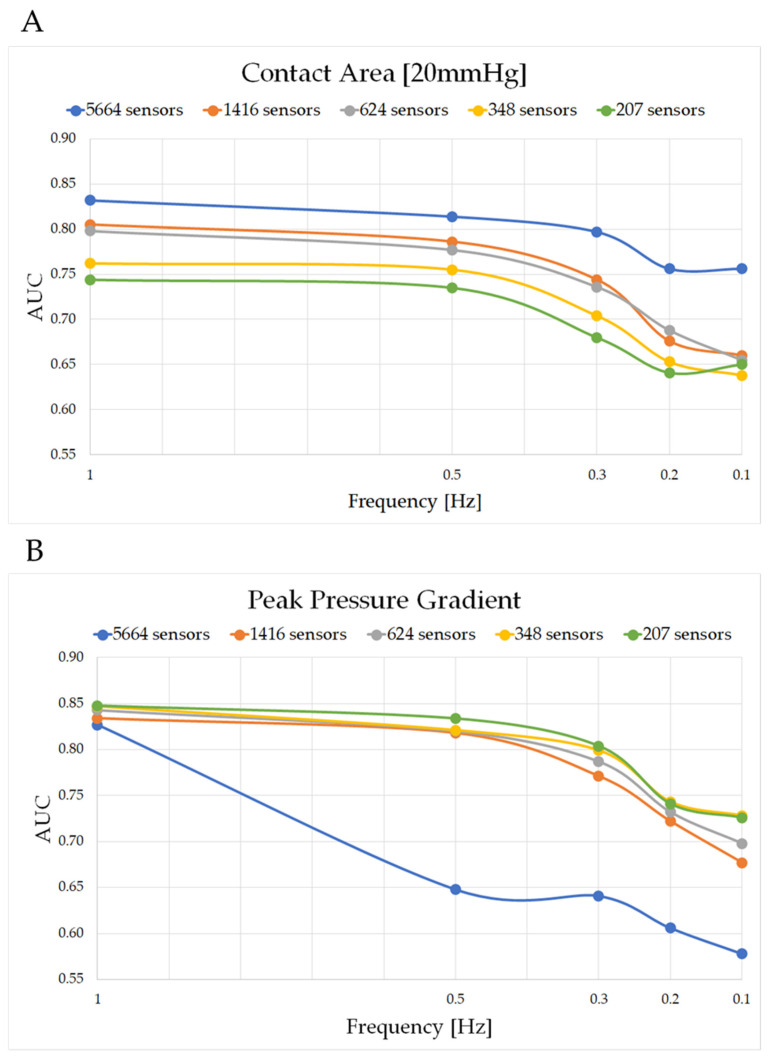
Trend of AUC at different spatial and temporal resolutions for (**A**) contact area (≥ 20 mmHg) and (**B**) peak pressure gradient estimated from air cell mattress. Each marker represents an AUC value at a specific sampling frequency, and the different colors represent a different spatial resolution, with blue depicting the original number of 5664 sensors.

Closer examination of the data revealed that pressure parameters followed two general trends in their spatial resolution, at each sampling frequency. Peak pressure and peak pressure gradient showed higher AUC values for a lower number of sensors, as opposed to the COP and contact area, which revealed the opposite trend. Figure 4A,B show a comparison between the contact area (≥ 20 mmHg) and peak pressure gradient, estimated on the air cell mattress. The former showed the highest AUC values at the original resolution of 5664 sensors for all sampling frequencies (AUC = 0.83 at 1 Hz and 0.76 at 0.1 Hz). By contrast, the latter showed the highest AUC values at a spatial resolution of 207 sensors, with AUC equal to 0.83 at 1 Hz and 0.58 at 0.1 Hz. It was interesting to note that, at the original spatial resolution, AUC dramatically reduced by approximately 22% for sampling frequencies ≥ 0.5 Hz.

It was interesting to note that, for each sampling frequency, the AUC associated with COP in both longitudinal and transverse directions showed little variations when spatial resolution was decreased, on the foam mattress. On the air cell, the original spatial resolution was associated with higher AUC values when compared to lower resolutions, which were associated with little variations.

### 3.2. Posture Classification: Convolutional Neural Network

Table 4 shows the total accuracy across all participants of the CNN in classifying the static postures adopted on both the foam and air cell mattress, for each of the spatial resolution. The results revealed that, on the air cell mattress down sampling, the spatial resolution resulted in a similar accuracy in postures classification (84−87%) when prediction was compared to the original resolution of 5664 sensors (86%). By contrast, on the foam mattress, the accuracy decreased by ~10% when the number of sensors was reduced at <350 sensors. A closer examination of the data revealed that prediction of static postures was more accurate when postures were performed on the air cell mattress, for all the resolutions.

## 4. Discussion

The present study performed a sensitivity analysis to optimize spatial and temporal configurations of a pressure sensing array to detect mobility events and predict static postures adopted on two mattress systems (Figure 2 and Table 2). Our findings revealed that high levels of accuracy in detecting posture and mobility events could be achieved with reduced array of sensing points, sampled at lower frequencies. Down sampling the spatial resolution increased the predictive ability of some parameters, e.g., peak pressure and peak pressure gradient at all sampling frequencies (Figure 4B). By contrast, the COP and contact area had reduced predictive performance, although the decrements were relatively low (1−10%) (Table 2).

Posture detection with the CNN showed an approximately similar accuracy at the different spatial resolution. Prediction on the air cell mattress resulted in an accuracy > 80% at all the spatial resolutions. Accuracy on the foam mattress was found to be 71% for > 350 sensors, with this value decreasing when the number of sensors was reduced (Table 4).

These studies using CNN thus demonstrated that a high accuracy in posture classification can be achieved at lower spatial resolution. Indeed, an accuracy of 90% in predicting lying postures was achieved using a pressure sensing array with 171 sensors placed in a 19 × 9 grid [16]. Moreover, an accuracy of 82% was obtained with a system of 64 sensors placed in a 8 × 8 grid [17]. It is evident that the present findings are comparable with previous studies, with our results showing a similar accuracy in classifying postures with CNN when lower spatial resolutions are compared with systems with a high number of sensors (Table 4).

Our results also revealed a difference in predictive accuracy when mattress conditions were compared (Table 3 and Table 4). This could be explained by the different nature of the lateral postures performed on the foam and air cell mattress, which might have influenced the predictive ability of both pressure parameters and the CNN. In addition, this could also have been influenced by the different characteristics of the two mattresses [18], with the air cell mattress resulting in more immersion when compared to the foam mattress. This implies an increase in the contact area, whose predictive ability in detecting postural change events is higher when compared to its counterpart on the foam mattress.

Our study proposed an optimization process which involved down sampling the number of sensors and frequency of data acquisition. The former involved a series of coarser configurations obtained by combining regions of sensors, e.g., 2 × 2, 3 × 3, and averaging their pressure values. This method is reflected in previous studies assessing the optimal sensor distribution in sitting [13]. They reported that 19 sensors, optimally distributed at the seat and back interface, produced the same accuracy of 82% in predicting static postures when compared to 4032 sensors [13]. However, they limited their analysis to sitting environment and to evaluating the accuracy by only predicting static postures against different spatial resolutions. By contrast, another recent study [14] explored a range of frequencies of acquisition at a spatial resolution of 5564 sensors. It utilized correlation coefficients between pressure values from 5564 sensor cells at the established time frames and thresholds to identify postural change events in lying. They reported that the number of postural change events was comparable with the original temporal resolution for a sampling frequency equal to 60 s (0.017 Hz). This is in contrast with our results where, at the same spatial resolution of 5664 sensors, 1 Hz shows the highest predictive ability.

The present study has several limitations. The experimental studies involved a relatively young, able-bodied cohort, and this precludes generalizing the findings to all specific sub-populations deemed to be at risk of developing pressure ulcers, i.e., the elderly, spinal cord injured, and those individuals in intensive care units. Further limitations involved a pre-determined order of postural changes following a relatively short period of 20 min in which each posture was maintained. In addition, the different natures of the lateral postures performed on the foam and air cell mattresses influenced the predictive ability of the pressure parameters in detecting postural change events and the CNN in predicting static postures. Indeed, the manual lateral turning performed on the air cell mattress resulted in higher prediction of movement and postural discrimination. By contrast, the automatic lateral tilting on the foam mattress had a lower predictive outcome, whereby there is smaller change in the distribution of pressure values during this movement [19]. Indeed, misclassification was observed to occur in the lateral posture classification. In addition, this precludes a full comparison of the mattress conditions.

Continuous pressure monitoring has been proven to provide promising indicators of posture and mobility when integrated with intelligent algorithms. The present study demonstrated that reducing the number of sensors and acquisition frequency influences the predictive ability of pressure parameters. They provide different clinically relevant pieces of information to clinicians, carers, and patients on posture, mobility, and PU risk. Therefore, the optimal spatial and temporal resolution would be a composite of the individual parameters’ predictive ability. In the light of our findings, we demonstrated that the high-resolution systems can be down sampled in the number of sensors and sampling frequency, while still maintaining a similar predictive ability in detecting postural change events and predicting static postures. This technological improvement could be utilized to help (i) reduce the volume and redundancy of data, decreasing computational costs (this will lead to a more efficient data mining and processing to achieve clinically meaningful parameters and provide efficient feedback); (ii) lower costs of manufacture, which will lead to more accessibility to diverse clinical settings and range of patients. Further studies are required to establish how new sensing systems could be developed and what resolution is needed to support clinical decision making. In addition, integrating IoT could support a more efficient PU prevention through real-time feedback and diagnosis, of which remote and community care would benefit.

## 5. Conclusions

This study has demonstrated that accurate detection of posture and mobility events can be achieved with pressure sensing array with a relatively low number of sensors and sampling rate. Our optimization process, involving both spatial and temporal domains, resulted in equivalent performance to high-resolution pressure sensing arrays. These findings are novel and important as they highlight the potential for our approach to optimize commercial systems and reduce their cost, data redundancy, and complexity. This will support clinical translation and utility in a variety of settings, including in the community.

## Figures and Tables

**Table 1:** Summary of the anthropometric characteristics (mean ± std) of participants involved in each study.

	Age [yo]	Height [m]	Weight [kg]	BMI [kg/m^2^]
Foam mattress	33 ± 6.71 (range 27−56)	1.72 ± 0.1	70.3 ± 15.9	23.6 ± 3.4 (range 19−30)
Air cell mattress	34.4 ± 11.6 (range 21−69)	1.71 ± 1.0	73.1 ± 18.3	24.5 ± 4.2 (range 19−30)

**Table 2:** Combination of spatial and temporal resolutions obtained by down sampling the original configuration of 5564 sensors sampling at 1 Hz. This results in a lower number of sensors and higher acquisition frequencies.

N Sensors	Sampling Frequency				
5664	1 Hz	0.5 Hz	0.3 Hz	0.2 Hz	0.1 Hz
1416					
624					
348					
207					

**Table 3:** AUC of the pressure parameters for the combinations of spatial and temporal configurations.

	AUC Values									
	Sampling frequency									
	1 Hz	0.5 Hz	0.3 Hz	0.2 Hz	0.1 Hz	1 Hz	0.5 Hz	0.3 Hz	0.2 Hz	0.1 Hz
	Foam mattress					Air cell mattress				
N sensors	COP–longitudinal direction									
5664	0.71	0.70	0.70	0.68	0.65	0.88	0.86	0.83	0.81	0.78
1416	0.71	0.70	0.70	0.68	0.65	0.83	0.80	0.78	0.74	0.69
624	0.71	0.70	0.70	0.67	0.64	0.83	0.80	0.78	0.73	0.68
348	0.71	0.70	0.70	0.68	0.65	0.83	0.80	0.78	0.74	0.69
207	0.71	0.70	0.70	0.68	0.65	0.83	0.79	0.78	0.74	0.68
	COP–transverse direction									
5664	0.67	0.69	0.68	0.67	0.64	0.86	0.84	0.82	0.78	0.78
1416	0.67	0.68	0.68	0.67	0.64	0.81	0.79	0.76	0.73	0.70
624	0.67	0.68	0.68	0.66	0.63	0.81	0.79	0.76	0.73	0.70
348	0.67	0.68	0.68	0.66	0.63	0.81	0.79	0.77	0.73	0.68
207	0.67	0.69	0.68	0.66	0.63	0.81	0.79	0.76	0.73	0.69
	Contact Area [20 mmHg]									
5664	0.69	0.67	0.67	0.65	0.61	0.83	0.81	0.80	0.76	0.76
1416	0.65	0.66	0.66	0.63	0.63	0.81	0.79	0.74	0.68	0.66
624	0.66	0.67	0.67	0.64	0.62	0.80	0.78	0.74	0.69	0.66
348	0.64	0.65	0.65	0.63	0.59	0.76	0.76	0.70	0.65	0.64
207	0.63	0.64	0.64	0.62	0.59	0.74	0.74	0.68	0.64	0.65
	Peak pressure									
5664	0.62	0.63	0.63	0.61	0.58	0.82	0.80	0.78	0.73	0.68
1416	0.63	0.64	0.64	0.62	0.58	0.85	0.84	0.81	0.77	0.73
624	0.66	0.66	0.67	0.64	0.60	0.86	0.84	0.82	0.77	0.74
348	0.66	0.67	0.68	0.67	0.62	0.87	0.84	0.83	0.78	0.76
207	0.67	0.67	0.68	0.66	0.62	0.87	0.85	0.84	0.78	0.76
	Peak pressure gradient									
5664	0.63	0.62	0.63	0.63	0.59	0.83	0.65	0.64	0.61	0.58
1416	0.62	0.63	0.63	0.61	0.58	0.83	0.82	0.77	0.72	0.68
624	0.63	0.64	0.63	0.62	0.58	0.84	0.82	0.79	0.73	0.70
348	0.65	0.65	0.66	0.62	0.58	0.85	0.82	0.80	0.74	0.73
207	0.64	0.67	0.66	0.64	0.61	0.85	0.83	0.80	0.74	0.73

**Table 4:** Total accuracy [%] in posture classification at different spatial resolutions for postures adopted on foam and air cell mattress.

	Total Accuracy [%]	
N Sensors	Foam Mattress	Air Cell Mattress
5664	71	86
1416	71	88
624	71	84
348	63	87
207	63	84

## Data Availability

Data sharing not applicable.
